# Postpartum Thyroid Dysfunction in Women With Known and Newly Diagnosed Hypothyroidism in Early Pregnancy

**DOI:** 10.3389/fendo.2021.746329

**Published:** 2021-11-26

**Authors:** Xiaotong Gao, Xichang Wang, Yutong Han, Haoyu Wang, Jiashu Li, Yuanyuan Hou, Yang Yang, Huiru Wang, Weiping Teng, Zhongyan Shan

**Affiliations:** Department of Endocrinology and Metabolism, Institute of Endocrinology, National Health Commission (NHC) Key laboratory of Diagnosis and Treatment of Thyroid Diseases, The First Affiliated Hospital of China Medical University, Shenyang, China

**Keywords:** hypothyroidism, early pregnancy, postpartum thyroiditis, thyroid-stimulating hormone, levothyroxine

## Abstract

**Background:**

Hypothyroidism in the first trimester of pregnancy (T1) has great adverse effects on mothers and foetuses. However, few studies have investigated the influence on postpartum thyroid dysfunction. This study aimed to evaluate their long-term effect on postpartum thyroid function within one year after delivery.

**Methods:**

In total, 151 women were recruited from 1496 participants and were classified as newly diagnosed subclinical hypothyroidism (SCH) in T1 (ND-SCH, n=50), previously known SCH before pregnancy (PK-SCH, n=51) and previously known overt hypothyroidism (PK-OH, n=50). Their thyroid functions were dynamically monitored from pre-conception to one-year postpartum.

**Results:**

During pregnancy, the first thyroid functions’ test time in T1 were 5-8 gestational weeks. After delivery, the prevalence of postpartum thyroiditis (PPT) was comparable in women with previously known and newly diagnosed hypothyroidism [ND-SCH 62.0% *vs* PK-SCH 64.7% *vs* PK-OH 64.0%, P=0.96]. For the ND-SCH group, PPT was significantly related with thyroid-stimulating hormone (TSH) >4.0 mU/L occurring at <8 gestational weeks [OR=8.06, 95% CI, 2.08-31.29] and TSH levels outside 1.0-2.5 mU/L near childbirth [OR=3.73, 95% CI, 1.04-13.41]. For patients with known hypothyroidism before pregnancy (PK-SCH and PK-OH), TSH>2.5 mU/L in T1 [OR=3.55, 95% CI, 1.43-8.81] and TPOAb≥300 μIU/mL [OR=6.58, 95% CI, 2.05-21.12] were associated with PPT. Regardless of whether SCH was diagnosed before pregnancy or in T1, the levothyroxine (LT4) treatment was discontinued at delivery. More than 50% of the patients had to face the hypothyroidism phase of postpartum and restarted LT4 treatment in the first-year follow-up. The logistic regression analysis revealed that TSH elevation occurring at <8 gestational weeks [OR=2.48, 95% CI, 1.09-5.6], TSH levels outside 1.0-2.5 mU/L near childbirth [OR=3.42, 95% CI, 1.45-8.05], and TPOAb≥300 μIU/mL [OR=6.59, 95% CI, 1.79-24.30] were the risk factors.

**Conclusion:**

TSH elevation at <8 gestational weeks was associated with PPT after delivery in women with known and newly diagnosed hypothyroidism. Especially for SCH patients who stopped LT4 treatment at delivery, unsatisfactory TSH level at <8 gestational weeks and near childbirth, TPOAb≥300 μIU/mL were the risk factors for LT4 retreatment in one-year postpartum.

## Introduction

Hypothyroidism in pregnancy, even subclinical hypothyroidism (SCH), is associated with an increased risk of adverse pregnancy outcomes (1–4). The maternal requirement for thyroid hormones is enhanced during pregnancy (5–7), and begins at 4-6 gestational weeks (8). Endogenous thyroid hormone variation is affected by residual thyroid tissue and hormone synthesis capacity, human chorionic gonadotropin (hCG), thyroxine-binding globulin, thyroid peroxidase antibody (TPOAb)/thyroglobulin antibody (TgAb), iodine nutrition and other factors (9–12). However, in the condition of hypothyroidism, the sensitivity of thyroid function to hCG stimulation is reduced. Therefore, supplementation with exogenous thyroid hormones to meet maternal and foetal needs is critical for pregnancy, especially during early pregnancy.

According to the 2017 American Thyroid Association (ATA) guidelines, 4.0 mU/L of thyroid-stimulating hormone (TSH) could be regarded as the upper limit cut-off point for the diagnosis of SCH during pregnancy (13). Among all hypothyroidism patients, TSH<2.5 mU/L should be regarded as the control target. Since pregnancy can lead to an increased demand for levothyroxine (LT4), after delivery, pre-pregnancy LT4 isodose should be restored in patients with overt hypothyroidism (OH) diagnosed before pregnancy. Patients with SCH newly diagnosed during pregnancy are candidates for discontinuing LT4. The TSH levels should be evaluated at 6 weeks postpartum (13).

Postpartum thyroiditis (PPT) is an irregular thyroid condition that occurs within one year after delivery (14). The prevalence of PPT in the general population is 1-22% (15). Among women with hypothyroidism, especially those with autoimmune thyroiditis (AIT), the prevalence reached to 62.1-68.0% (16–18). However, few studies have reported the prevalence of PPT among women with SCH. In one study, 24.6% of patients with SCH diagnosed at 28 gestational weeks showed a sustained TSH elevation at 4.9 years postpartum (19). In another study, SCH was diagnosed at 22 gestational weeks, and 38.9% of women still maintained hypothyroidism at 7-19 months (20).

However, few studies focused on the postpartum prognosis of thyroid function when hypothyroidism occurs during early pregnancy. The aim of our study was to investigate the outcome of thyroid dysfunction within one year after delivery in women with thyroid dysfunction detected in the first trimester of gestation (both as newly diagnosed and previously known hypothyroidism).

## Materials and Methods

### Participants and Study Design

The investigators performed a chart review of 1496 pregnant women in the First Affiliated Hospital of China Medical University during a ten-year period (2010-2020), and the study was approved by the hospital ethics committee. All candidates signed informed consent forms and completed the questionnaire survey. They had been tested thyroid function routinely before pregnancy and had been treated once they were diagnosed with hypothyroidism. On the basis of their pre-pregnancy thyroid function, 409 women were diagnosed with hypothyroidism prior to conception from 2010 to 2020, and 108 women were consistently euthyroid pre-pregnancy but developed SCH in early pregnancy from 2017 to 2020, which was defined as TSH>4.0 mU/L with normal FT4 levels according to the 2017 ATA guidelines (13).

For the purposes of our study, we only included women who strictly met the following inclusion criteria: ① serum TSH, free triiodothyronine (FT3) and free thyroxine (FT4) monitored within 6 months before pregnancy (BP); in early (T1: 0 to 13^+6^ weeks), middle (T2: 14 to 27^+6^ weeks) and late (T3: 28 to 40 weeks) pregnancy; and in the first (A1: 6 weeks to 3 months), second (A2: 3 to 6 months) and third (A3: 6 months to 1 year) stages after delivery; ② serum TPOAb and TgAb monitored in T1; ③ complete and detailed clinical data covered by the questionnaire, i.e., age at the current pregnancy; pre-pregnancy body mass index (BMI); gestational weight gain (GWG, weight at delivery minus pre-pregnancy weight) (21); family history of thyroid disease; delivery history; abortion history; and LT4 replacement before, during and after pregnancy; ④ no history of diseases or medications affecting thyroid function (except for LT4 and progesterone); and ⑤ living in an iodine-sufficient area.

**Figure 1:**
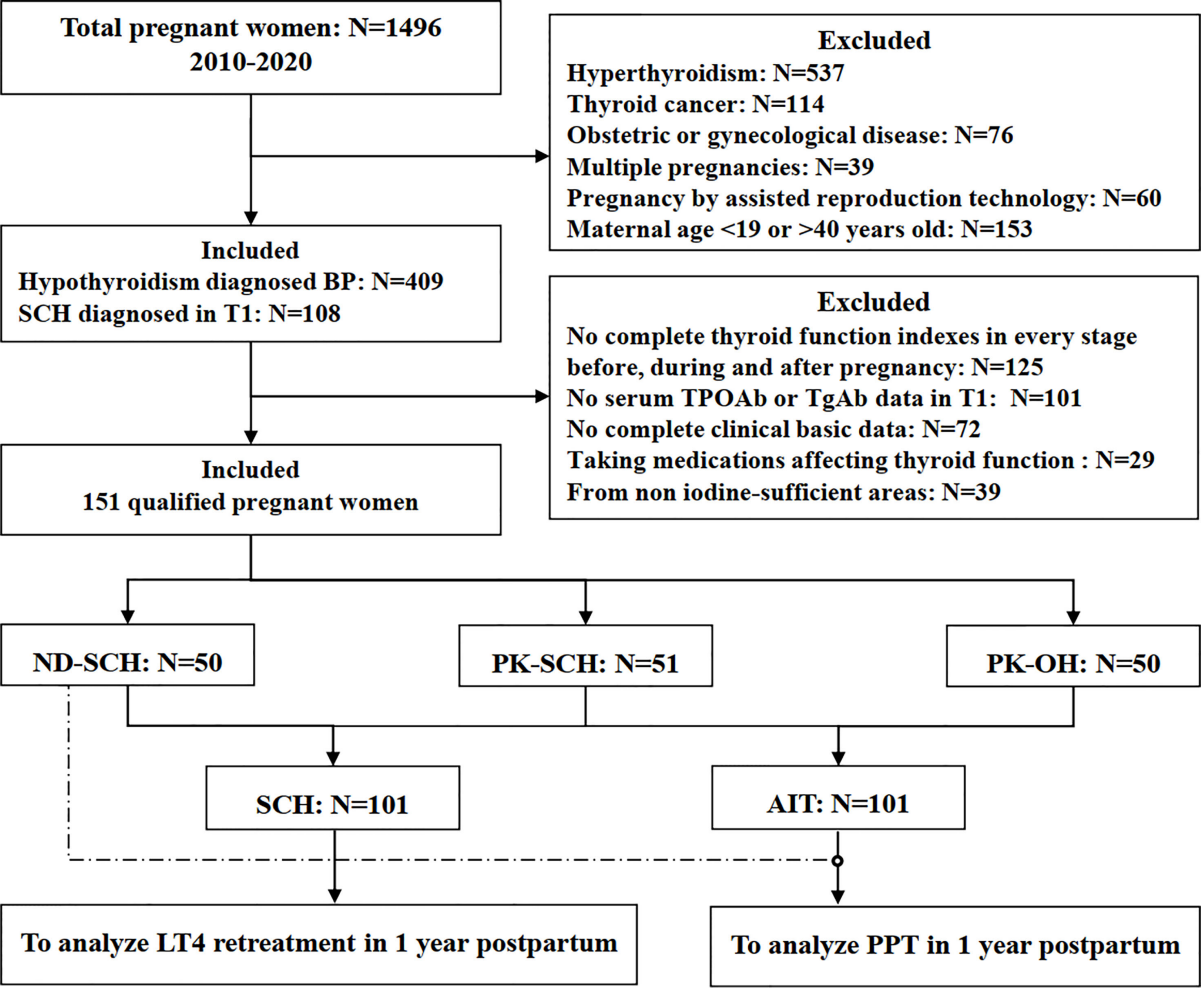
Flow chart of the study population. BP, before pregnancy; T1, early pregnancy; TPOAb, thyroid peroxidase antibody; TgAb, thyroglobulin antibody; SCH, subclinical hypothyroidism; ND-SCH, subclinical hypothyroidism newly diagnosed in early pregnancy; PK-SCH, previously known subclinical hypothyroidism before pregnancy; PK-OH, previously known autoimmune overt hypothyroidism; AIT, autoimmune hypothyroidism diagnosed before pregnancy; PPT, postpartum thyroiditis.

Based on the above criteria, in total, 151 women were recruited (**Figure 1**). According to the diagnosis time, disease degree and aetiology of hypothyroidism, the patients were further classified as follows: SCH newly diagnosed in the first trimester of pregnancy (ND-SCH, n=50); Previously known autoimmune SCH (PK-SCH, n=51); and previously known autoimmune overt hypothyroidism(PK-OH, n=50). All women with previously known hypothyroidism received adequate LT4 replacement within 6 months before pregnancy. The pregnant women in the ND-SCH group started LT4 intervention once they were diagnosed (22). The woman’s thyroid function was evaluated within 8 gestational weeks, followed by monitoring every 4-6 weeks to adjust the LT4 dose. The serum TSH target was maintained between the lower limit of the pregnancy-specific reference range and 2.5 mU/L throughout pregnancy.

The pregnant women with previously known and newly diagnosed SCH discontinued LT4 treatment after delivery. The pregnant women with PK-OH continued LT4 treatment after delivery, and the LT4 dose was adjusted according to the dose before delivery.

### Definition of Postpartum Thyroiditis

Among the SCH patients, PPT was diagnosed based on the occurrence of any of the following three possibilities within the first year after delivery ① transient thyrotoxicosis (TSH<0.27 mU/L, which is the lower limit of reference range in nonpregnant women) accompanied by transient hypothyroidism (TSH>4.20 mU/L, which is the upper limit of reference range in nonpregnant women) (biphasic PPT); ② only transient thyrotoxicosis; or ③ only transient hypothyroidism. Among the PK-OH patients, ‘only transient thyrotoxicosis’ and ‘only transient hypothyroidism’ were probably due to a surplus or deficit in the LT4 intervention, respectively. Therefore, PPT was diagnosed only in the following two cases: ① when the optimal postpartum LT4 dose was chosen, there was an occurrence of hypothyroidism conversed by transient thyrotoxicosis in the first postpartum year; or ② under the appropriated LT4 treatment after delivery, although the thyroid function was normal at 6 postpartum weeks, there were a remarkable thyroid dysfunction in the next 3-12 postpartum months.

### Measurement of Thyroid Function and Thyroid Autoimmunity

Thyroid function indicators were measured by electrochemiluminescence immunoassay system assay (Cobas; Roche Diagnostics, Basel, Switzerland). The reference ranges for nonpregnant women are as follows: FT3 3.1-6.8 pmol/L; FT4 9.0-22.0 pmol/L; TSH 0.27-4.20 mU/L; TPOAb<34 μIU/mL; and TgAb<115 μIU/mL. There were no changes in testing methodology during our study period. The gestational trimester-specific reference intervals tested in our laboratory were applied in the whole period of gestation (23). The intra- and interassay variation coefficients of the above variations were less than 10%. In our study, TPOAb≥300 μIU/mL indicates TPOAb positivity, and TgAb≥300 μIU/mL indicates TgAb positivity (24).

### Statistical Analysis

SPSS statistics for Windows v20.0 (SPSS, Inc., Chicago, IL) was used for the statistical analysis. Continuous variables are described as the mean ± standard deviation (M ± SD) or median and quartile difference (IQR), and categorical variables are described as numbers and corresponding percentages.

For continuous variables, one-way ANOVA or the Kruskal-Wallis test was utilized to compare the baseline characteristics of multiple groups, and *post hoc* Bonferroni correction was performed. For categorical variables, the chi-square test, Pearson’s chi-square test or Fisher’s exact test was used to compare frequencies.

A logistic regression model was used to estimate the prevalence of PPT and the prevalence of postpartum LT4 retreatment, to analyse the correlation with multiple variables and confounding factors. Maternal age and family history of thyroid disease were controlled as covariates (25). The results were presented as odds ratios (ORs), 95% confidence intervals (95%CIs) and significance. P<0.05 was considered statistically significant.

## Results

### Clinical Characteristics of Pregnant Women With Different Types of Hypothyroidism

The clinical characteristics of the ND-SCH, PK-SCH, and PK-OH groups are shown in **Table 1**. There were no significant differences in most baseline clinical data, namely, maternal age, pre-pregnancy BMI, the proportion of GWG>15 kg (21), family history of thyroid disease, abortion history among the three groups.

**Table 1:** Descriptive statistics of women grouped according to thyroid function (n = 151).

	*ND-SCH*	*PK-SCH*	*PK-OH*	*P*
• *Clinical basic data*				
*N*	50	51	50	*0.990*
*Maternal age (years)*	30.98 ± 4.00	30.00 ± 3.09	31.02 ± 4.26	*0.315*
*Pre-pregnancy BMI (kg/m2)*	21.19 ± 3.04	22.55 ± 3.70	22.02 ± 2.99	*0.111*
*GWG≥15 kg, n (%)*	27 (54.0)	25 (49.0)	27 (54.0)	*0.845*
*Family history of thyroid disease, n (%)*	10 (20.0)	5 (9.8)	8 (16.0)	*0.356*
*Abortion history, n (%)*	15 (30.0)	16 (31.4)	16 (32.0)	*0.976*
• *Thyroid function and thyroid autoantibodies*				
*I. The period before pregnancy (BP)*				
FT4 (pmol/L)*	11.27 (10.34-12.99)^bc^	16.38 (14.96-18.74)^ac^	13.48 (11.68-15.05)^ab^	*0.000*
FT3 (pmol/L)*	3.87 (3.67-4.36)^c^	4.13 (3.81-4.50)	4.20 (3.79-4.53)^a^	*0.035*
* TSH (mU/L)*	1.65 (1.24-2.56)	2.18 (1.03-3.19)	2.00 (0.74-3.17)	*0.089*
*II. The first trimester of pregnancy (T1)*				
Gestational age (weeks)*	6.6 (5.0-12.0)^bc^	6.0 (5.0-7.0)^a^	6.0 (5.0-7.1)^a^	*0.000*
FT4 (pmol/L)*	13.26 (11.84-15.98)^bc^	16.24 (14.67-18.42)^a^	14.67 (13.33-17.13)^a^	*0.000*
* FT3 (pmol/L)*	4.29 (3.75-4.55)	4.17 (3.71-4.61)	4.26 (3.99-4.58)	*0.692*
TSH (mU/L)*	5.56 (4.39-7.12)^bc^	2.78 (1.73-3.63)^a^	2.66 (1.95-4.14)^a^	*0.000*
TPOAb≥300 μIU/mL, n (%)*	10 (20.0)	14 (27.5)	24 (48.0)	*0.008*
* TgAb≥300 μIU/mL, n (%)*	13 (26.0)	16 (31.4)	16 (32.0)	*0.771*
* TPOAb/TgAb +, n (%)*	19 (38.0)	22 (43.1)	27 (54.0)	*0.260*
*III. The second trimester of pregnancy (T2)*				
* Gestational age (weeks)*	20.1 (19.0-21.3)	20.0 (19.0-21.0)	20.0 (19.0-21.5)	*0.822*
FT4 (pmol/L)*	12.03 (11.10-13.71)^b^	13.50 (11.50-15.10)^a^	12.58 (10.85-14.23)	*0.039*
FT3 (pmol/L)*	4.03 (3.63-4.45)	4.08 (3.37-4.54)^c^	4.22 (3.85-4.72)^b^	*0.031*
TSH (mU/L)*	1.86 (1.28-3.13)^c^	1.78 (1.17-2.51)	1.30 (0.75-2.07)^a^	*0.009*
*IV. The third trimester of pregnancy (T3)*				
* Gestational age (weeks)*	35.0 (33.0-36.3)	35.0 (32.0-37.0)	35.0 (33.0-37.0)	*0.831*
FT4 (pmol/L)*	11.27 (10.18-13.34)	12.71 (10.53-13.74)^c^	11.27 (10.23-12.74)^b^	*0.020*
* FT3 (pmol/L)*	3.89 (3.47-4.18)	4.09 (3.68-4.37)	4.08 (3.58-4.44)	*0.137*
* TSH (mU/L)*	1.56 (0.94-2.53)	1.65 (1.05-2.55)	1.36 (0.81-2.00)	*0.273*
• *Postpartum thyroid function status*				
*Euthyroid, n (%)*	18 (36.0)	17 (33.3)	15 (30.0)	*0.815*
*Abnormal thyroid function, n (%)*	32 (64.0)	34 (66.7)	35 (70.0)	*0.815*
*PPT, n (%)*	31 (62.0)	33 (64.7)	32 (64.0)	*0.958*
Only transient hypothyroidism, n (%)*	23 (46.0)^c^	25 (49.0)^c^	2 (4.0)^ab^	*0.000*
*Only transient thyrotoxicosis, n (%)*	3 (6.0)	3 (5.9)	1 (2.0)	*0.254*
*Total hypothyroidism phase, n (%)*	28 (56.0)	30 (58.8)	34 (68.0)	*0.437*
*Hyperthyroidism, n (%)*	1 (2.0)	1 (2.0)	——	*0.989*

Data were presented as the median (interquartile range: 25-75%), M ± SD or n (%) as appropriate.

*presented as P<0.05; ^a^presented that there was significant difference in the group with the ND-SCH group, P<0.05; ^b^presented that there was significant difference in the group with the PK-SCH group, P<0.05; ^c^presented that there was significant difference in the group with the PK-OH group, P<0.05.

ND-SCH, subclinical hypothyroidism newly diagnosed in early pregnancy; PK-SCH, previously known subclinical hypothyroidism; PK-OH, previously known autoimmune overt hypothyroidism; TSH, thyroid stimulating hormone; FT3, free triiodothyronine; FT4, free thyroxine; TPOAb, thyroid peroxidase antibody; TgAb, thyroglobulin antibody; BMI, body mass index; GWG, gestational weight gain; PPT, postpartum thyroiditis.

The follow-up time points were as followed: BP, 5.39 ± 1.84 months; T1, 6.72 ± 2.11 gestational weeks; T2, 20.38 ± 1.73 gestational weeks; T3, 34.93 ± 2.29 gestational weeks; the first stage after delivery (A1), 8.13± 2.69 weeks; the second stage after delivery (A2), 5.62± 2.04 months; the third stage after delivery (A2), 10.47± 4.36 months. Thyroid function indicators were measured by Roche, and during pregnancy, the trimester-specific reference intervals tested in our laboratory were applied (23).

Among the women with ND-SCH, the gestational age at SCH diagnosis was 6.6 (5.0-12.0) weeks, and the initial TSH level was 5.56 (4.39-7.12) mU/L. Women with previously known hypothyroidism already received adequate LT4 replacement before conception, and their TSH levels were maintained below 2.5 mU/L as shown in **Table 1**. During pregnancy, the first monitoring time in T1 was within 8 gestational weeks, and no differences were observed among these three groups. In the whole cohort, LT4 adjustments were applied individually to maintain TSH<2.5 mU/L until birth [ND-SCH 1.56 (0.94-2.53) mU/L, PK-SCH 1.65 (1.05-2.55) mU/L, PK-OH 1.36 (0.81-2.00) mU/L]. After delivery, thyroid function was tested at least three times [A1: 8.13 ± 2.69 weeks, A2: 5.62 ± 2.04 months and A3: 10.47 ± 4.36 months].

**Figure 2:**
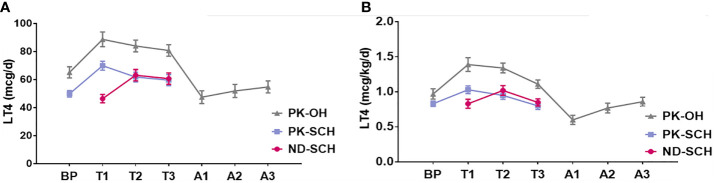
LT4 dose adjustment before, during and after pregnancy. The whole pregnant women were followed up in every stage before, during and after pregnancy. Pregnant women with SCH stopped taking LT4 once they gave birth; As for pregnant women with PK-OH, LT4 dose were adjusted according to the dose before childbirth. Then adjusted LT4 dose accordingly in the light of the thyroid function postpartum. **(A)** shows the variations of daily LT4 dose (mcg/d) in each group and **(B)** shows weight adjusted LT4 dose (mcg/kg/d) in each group.

As shown in **Figure 2**, the LT4 dose was remarkably correlated with the impairment degree of hypothyroidism. The LT4 doses in the PK-OH group were obviously higher than those in the ND-SCH and PK-SCH groups. However, regardless of the cause of hypothyroidism, the LT4 dose first presented an increasing trend, followed by a decreasing change in the following progression of gestation. In late pregnancy, the LT4 dose returned to the initial dose in early pregnancy in the ND-SCH group and the original pre-pregnancy dose in the other two groups [initial dose *vs* final dose: ND-SCH 0.83 ± 0.43 mcg/kg/d *vs* 0.85 ± 0.34 mcg/kg/d, PK-SCH 0.83 ± 0.27 mcg/kg/d *vs* 0.80 ± 0.37 mcg/kg/d, PK-OH 0.97 ± 0.49 mcg/kg/d *vs* 1.11 ± 0.39 mcg/kg/d, P>0.05].

### Outcomes and Influencing Factors of PPT in ND-SCH Women

After delivery, LT4 treatment was suspended in the ND-SCH group. During the following one year, 32 (64.0%) patients developed thyroid dysfunction, including 31 (62.0%) cases of PPT and 1 (2.0%) case of hyperthyroidism (**Table 1**). A comparison of the clinical characteristics of the postpartum-euthyroid group (n=18) and PPT group (n=31) showed that the gestational age at diagnosis in the postpartum-euthyroid group was apparently later than that in the PPT group [12.0 (6.4-14.9) weeks *vs* 6.0 (5.0-9.0) weeks, P=0.001]. The rate of postpartum-euthyroid women whose TSH level was controlled at 1.0-2.5 mU/L in late pregnancy was 1.72-fold greater than that in the PPT group [13/18 (72.2%) *vs* 13/31 (41.9%), P=0.041] (**Supplementary Table 1**).

The logistic regression analysis further confirmed that gestational SCH diagnosed at <8 weeks was a risk factor for developing PPT [OR=8.063, 95% CI, 2.078-31.285; P=0.003]. TSH levels outside the 1.0-2.5 mU/L range before childbirth were also a risk factor [OR=3.725, 95% CI, 1.035-13.411; P=0.044]. There were no apparent correlations between PPT and other adjusted factors (**Table 2**).

**Table 2:** Logistic regression model for the risk factors of developing PPT in women with ND-SCH (n = 50).

ND-SCH group	OR	95%CI		P
Maternal age (years)	0.966	0.835	1.117	0.638
Family history of thyroid disease (yes vs no)	2.783	0.521	14.866	0.231
Initial TSH level in T1 (mU/L)	1.128	0.911	1.398	0.269
SCH diagnosed < 8 weeks (yes vs no)*	8.063	2.078	31.285	0.003
TSH <1.0 mU/L or >2.5 mU/L in T3 (yes or no)*	3.725	1.035	13.411	0.044
TPOAb≥300μIU/mL (yes or no)	1.295	0.279	6.024	0.741
TgAb≥300μIU/mL (yes or no)	1.434	0.360	5.715	0.609

*P < 0.05. The crude OR for maternal age and family history of thyroid disease were calculated first and we found no apparent correlations between PPT and them. Subsequently, the OR value for the potential influencing factors were adjusted by these two basic factors.

TSH, thyroid-stimulating hormone; TPOAb, thyroid peroxidase antibody; TgAb, thyroglobulin antibody; PPT, postpartum thyroiditis; T1, the first trimester of pregnancy; T3, the third trimester of pregnancy.

### Outcomes and Influencing Factors of PPT in Women With Previously Known Hypothyroidism

After delivery, the PK-SCH women also discontinued LT4 treatment. Thirty-four (66.7%) patients developed thyroid dysfunction in the first year postpartum, including 33 (64.7%) cases of PPT and 1 (2.0%) case of hyperthyroidism. In the PK-OH group, 35 (70.0%) patients developed thyroid dysfunction, including 32 (64.0%) cases of PPT, 2 (4.0%) cases of transient hypothyroidism and 1 (2.0%) case of transient thyrotoxicosis (**Table 1**).

The main aetiology for both PK-SCH and PK-OH groups was AIT, and the prevalence of PPT was also similar [PK-SCH 33/51 (64.7%) *vs* PK-OH 32/50 (64.0%), P=0.941] (**Table 1**). Hence, these two groups were combined as the AIT group (n=101) for further analysis.

A comparison of the clinical characteristics of the postpartum-euthyroid group (n=32) and PPT group (n=65) showed that the rate of postpartum-euthyroid women with a TSH level>2.5 mU/L in T1 was obviously lower than that in the PPT group [11/32 (34.4%) *vs* 43/65 (66.2%), P=0.003]. The positive rate of TPOAb≥300 μIU/mL among the postpartum-euthyroid women was a quarter of that in the PPT group [4/32 (12.5%) *vs* 32/65 (49.2%), P=0.001] (**Supplementary Table 2**).

The logistic regression analysis also confirmed that TSH>2.5 mU/L in T1 was a risk factor for developing PPT [OR=3.551, 95% CI, 1.430-8.814; P=0.006], and TPOAb≥300 μIU/mL was another risk factor [OR=6.578, 95% CI, 2.049-21.122; P=0.002]. There were no apparent correlations between PPT and the other adjusted factors (**Table 3**).

**Table 3:** Logistic regression model for the risk factors of developing PPT in women with previously known hypothyroidism caused by AIT (n = 101).

AIT group	OR		95%CI		P
Maternal age (years)	0.967		0.864	1.082	0.559
Family history of thyroid disease (yes vs no)	3.056		0.635	14.707	0.164
TSH in T1 >2.5 mU/L (yes vs no)*	3.551		1.430	8.814	0.006
TSH <1.0 mU/L or >2.5 mU/L in T3 (yes or no)	0.619		0.259	1.476	0.279
TPOAb≥300μIU/mL (yes or no)*	6.578		2.049	21.122	0.002
TgAb≥300μIU/mL (yes or no)	2.005		0.744	5.407	0.169

*P < 0.05. The crude OR for maternal age and family history of thyroid disease were calculated first and we found no apparent correlations between PPT and them. Subsequently, the OR value for the potential influencing factors were adjusted by these two basic factors.

The main aetiology of previously known SCH and OH was autoimmune hypothyroidism, so combined these two groups to further analysis (PK-SCH & PK-OH).

TSH, thyroid stimulating hormone; TPOAb, thyroid peroxidase antibody; TgAb, thyroglobulin antibody; PPT, postpartum thyroiditis; T1, the first trimester of pregnancy; T3, the third trimester of pregnancy.

### Outcomes and Influencing Factors of LT4 Retreatment After Delivery in SCH Women

Regardless of whether transient thyrotoxicosis occurred during the first-year follow-up after delivery, once the hypothyroidism phase occurred, LT4 replacement was restarted to prevent a further decline in postpartum thyroid function in all SCH women. The prevalence of LT4 retreatment was comparable between the ND-SCH and PK-SCH groups [28/50 (56.0%) *vs* 30/51 (58.8%), P=0.774]. Hence, these groups were combined as the SCH group (n=101) for further analysis.

In the SCH group, there were 41 women completely stop LT4 replacement in the first year after delivery (LT4-discontinuation group), and 58 women restarted LT4 treatment due to the occurrence of hypothyroidism (LT4-retreatment group). A comparison of the clinical characteristics showed that the rate of women with a TSH level>2.5 mU/L within 8 gestational weeks in the LT4-retreatment group was 1.54-fold greater than that in the LT4-discontinuation group [37/58 (63.8%) *vs* 17/41 (41.5%), P=0.028]. The rate of women whose TSH level was controlled at 1.0-2.5 mU/L in late pregnancy in the LT4-retreatment group was three-fifth of that in the LT4-discontinuation group [24/58 (41.4%) *vs* 29/41 (70.7%), P=0.004], and the positive rate of TPOAb≥300 μIU/mL in the LT4-retreatment group was 4.96-fold greater than that in the LT4-discontinuation group [20/58 (34.5%) *vs* 3/41 (7.3%), P=0.002] (**Supplementary Table 3**).

The logistic regression analysis also confirmed that hypothyroidism occurring at <8 gestational weeks [OR=2.478, 95% CI, 1.087-5.648; P=0.031], TSH levels outside 1.0-2.5 mU/L near childbirth [OR=3.418, 95% CI, 1.451-8.049; P=0.005], and TPOAb≥300 μIU/mL [OR=6.589, 95% CI, 1.787-24.302; P=0.005] were the risk factors for LT4 retreatment at one year postpartum (**Table 4**).

**Table 4:** Logistic regression model for the risk factors of LT4 retreatment in all SCH women (n = 101).

SCH group	OR	95%CI		P
Maternal age (years)	0.999	0.894	1.118	0.991
Family history of thyroid disease (yes vs no)	1.500	0.472	4.772	0.492
Hypothyroidism within 8 weeks (yes vs no)*	2.478	1.087	5.648	0.031
TSH <1.0 mU/L or >2.5 mU/L in T3 (yes or no)*	3.418	1.451	8.049	0.005
TPOAb≥300μIU/mL (yes or no)*	6.589	1.787	24.302	0.005
TgAb≥300μIU/mL (yes or no)	1.526	0.619	3.763	0.358

*P < 0.05. The crude OR for maternal age and family history of thyroid disease were calculated first and we found no apparent correlations between postpartum LT4 retreatment and them. Subsequently, the OR value for the potential influencing factors were adjusted by these two basic factors.

Regardless of whether SCH was newly diagnosed in early pregnancy or previously known, they were combined as SCH group to further analysis (ND-SCH & PK-SCH).

TSH, thyroid stimulating hormone; TPOAb, thyroid peroxidase antibody; TgAb, thyroglobulin antibody; T1, the first trimester of pregnancy; T3, the third trimester of pregnancy.

## Discussion

In this retrospective study, 151 hypothyroid women were recruited to analyse the postpartum prognosis of thyroid function in the first year after delivery and the influencing factors during pregnancy. We found that among the ND-SCH women, when LT4 intake was stopped after delivery, the prevalence of PPT was 62.0% during the 12-months monitoring of thyroid function after delivery. The patients with SCH occurring at <8 gestational weeks or whose TSH levels were outside the range of 1.0-2.5 mU/L in late pregnancy were more likely to suffer from PPT; Women with PK-SCH also discontinued LT4 intake after delivery. However, both TSH>2.5 mU/L in T1 and TPOAb≥300 μIU/mL were the risk factors for developing PPT in one year postpartum in PK-SCH and PK-OH patients, and the prevalences were separately 64.7% and 64.0%; Regardless of whether SCH occurred before pregnancy or during early pregnancy, the prevalence of the postpartum hypothyroidism phase was similar (PK-SCH 58.8% *vs* ND-SCH 56.0%), leading to LT4 retreatment after delivery. By combining all these SCH women into a unified group, we found that unideal TSH elevation at <8 gestational weeks, TSH levels outside the range of 1.0-2.5 mU/L in late pregnancy and TPOAb≥300 μIU/mL were the risk factors for LT4 retreatment in the first postpartum year.

Early gestation is a time of rapid development of the central nervous system in the foetus. During this time, the mother is the only source of foetal thyroid hormones (7). Therefore, it is the most critical period for monitoring thyroid diseases during pregnancy. To date, no studies reported the influence of newly occurred SCH in early pregnancy on thyroid function postpartum, especially within 8 gestational weeks. And no studies assessed the influence of SCH occurred before or in early pregnancy on the LT4 strategy after delivery.

According to the 2017 ATA guidelines, if TSH >4.0 mU/L with normal serum T4, SCH could be diagnosed in early pregnancy and should start LT4 treatment, and the treatment might be considered to stop after delivery (13). In our study, the ND-SCH women who developed PPT in the first postpartum year had an earlier gestational week at diagnosis than those who did not develop PPT [6.0 (5.0-9.0) weeks *vs* 12.0 (6.4-14.9) weeks, P=0.001]. SCH appeared earlier, indicating that thyroid function was even worse during pregnancy, therefore, impaired thyroid function was difficult to recover after delivery. This phenomenon is consistent with another study conducted by Li et al. (20). Although the average gestational age of SCH in that study was 22 weeks, the patients who suffered from postpartum hypothyroidism had an earlier diagnosis time than those who did not [19 (14–26) weeks *vs* 22 (16–28) weeks, P<0.05]. In the long-term follow-up in our study, regardless of whether transient thyrotoxicosis occurred, the prevalence of the postpartum hypothyroidism phase was 56.0% in the ND-SCH group, which is higher than that in the previously mentioned study (38.9%). This finding also indicated that the recovery of thyroid function after delivery among SCH newly diagnosed patients in early pregnancy was worse than that of SCH patients diagnosed in middle pregnancy. Adequate LT4 replacement was a protective factor against PPT and controlling TSH levels at 1.0-2.5 mU/L in late pregnancy could reduce its occurrence. However, the TPOAb-positive rate in the ND-SCH group was only 20.0% in our study; therefore, AIT was not the main cause of the abnormal TSH levels. To date, mandatory universal salt iodization (USI) has been carried out in China for twenty years, and residents’ TSH levels are generally enhanced by an increase in iodine intake (26). All recruited women in our study were from Shenyang, which is an iodine-sufficient city. Therefore, iodine-induced nonautoimmune SCH was probably the main cause of ND-SCH.

Among known SCH and OH resulting from AIT, the overall prevalence of PPT was 65/101 (64.5%), which is similar to the prevalence reported in previous studies [Caixas et al. (16): 12/18 (66.7%); Galofre et al. (17): 18/29 (62.1%); Sergi et al. (18): 66/97 (68.0%)]. Stagnaro-Green indicated that more than 50% of TPOAb-positive women suffered from PPT after delivery, and the higher the TPOAb titre, the greater the risk of PPT (27). In our study, AIT hypothyroid women with TPOAb≥300 μIU/mL were more likely to develop PPT than those with TPOAb<300 μIU/mL [32/36 (88.9%) *vs* 33/61 (54.1%), P=0.000]. Similar to the results reported by Sergi et al., the prevalences of PPT among the TPOAb-positive and TPOAb-negative patients were 83% and 44%, respectively (18). Recently, Moleti et al. (28), found that three-quarters of PPT was biphasic in AIT-euthyroid women. Regarding AIT-hypothyroidism, the diagnostic criteria for PPT included only biphasic PPT, and the prevalence rate was only 18/98 (18.4%). The authors insisted that the euthyroid status in early pregnancy implied more viable thyroid tissue exposed to postpartum autoimmune attack. In contrast to the study focusing on biphasic PPT, our study found that TSH>2.5 mU/L in early pregnancy was a risk factor for the development of PPT in women with previously known AIT hypothyroidism. The prevalences of the only one-way hypothyroidism of PPT were 75% and 35% in the PK-SCH and PK-OH groups, respectively. TSH>2.5 mU/L implies that thyroid function deterioration might be more severe during early pregnancy and the LT4 replacement was not adequate; thus, thyroid function could be more prone to further decline postpartum and could not be restored to the pre-pregnancy levels. Therefore, more patients experienced the hypothyroidism phase of PPT.

In our study, we suspended the LT4 treatment after delivery, regardless of whether SCH was diagnosed before or during early pregnancy. The occurrence of the postpartum hypothyroidism phase is a signal for LT4 retreatment. We first compared the incidence of LT4 retreatment between the ND-SCH and PK-SCH groups and found no apparent difference. In total, approximately more than 50% of the SCH women eventually restarted LT4 replacement in the first year after delivery. During the first 8 gestational weeks, we considered both TSH>4.0 mU/L in the ND-SCH group and TSH>2.5 mU/L in the PK-SCH group as unideal TSH elevations in early pregnancy. And we found that these uncontrolled TSH elevations at < 8 gestational weeks was actually a risk factor to suffer from postpartum hypothyroidism and restarting LT4 replacement in the first postpartum year. Moreover, among SCH women, especially those with strong TPOAb positivity, should undergo frequent thyroid function evaluations after delivery to prevent the occurrence of postpartum hypothyroidism. In late pregnancy, the stimulating effect of hCG on thyroid function was decreased (29). During the time, TSH <1.0 mU/L may indicate the destruction of thyroid follicles with release of hormone (30), which would be one reason for SCH women being more likely to suffer hypothyroidism phase in postpartum. So that controlling TSH levels at 1.0-2.5 mU/L before delivery was advised.

Our study first focused on the influence of newly diagnosed SCH in early pregnancy on postpartum thyroid function. Regarding previously known SCH, they also discontinued LT4 treatment after delivery. Although the risk factors for the occurrence of PPT were differ, after combined analysis of these two types of SCH, we evaluated the shared risk factors for LT4 retreatment in the first year after delivery.

However, there are some limitations in our study. The sample size was small because the time points of detection were throughout the whole process, namely, before, during and after pregnancy, and the inclusion criteria were strict such that patient compliance would be limited. During our analysis, we selected women with a relatively high level of thyroid autoimmunity as TPOAb/TGAb positive and determined this as one of the risk factors in our study. All patients were regarded as having adequate iodine nutrition according to the local iodine-sufficient nutrition situation, and we did not detect urinary iodine. We look forward to large-scale studies in the future.

Overall, regardless of whether SCH is diagnosed before pregnancy or newly diagnosed during early pregnancy, uncontrolled TSH elevation within 8 gestational weeks is a shared risk factor for PPT and LT4 retreatment in the first year after delivery. Therefore, it is essential to monitor the thyroid function of pregnant women during the first 8 gestational weeks in early pregnancy and for them to undergo frequently thyroid function evaluations within one year postpartum to identify thyroid dysfunction in a timely manner and adjust the LT4 strategy accordingly.

## Data Availability Statement

The original contributions presented in the study are included in the article/**Supplementary Material**. Further inquiries can be directed to the corresponding author.

## Ethics Statement

The studies involving human participants were reviewed and approved by China Medical University (Ethics Committee of China Medical University [2012] 2011-32-4). The patients/participants provided their written informed consent to participate in this study.

## Author Contributions

We sincerely appreciate the strong support and cooperation of members responsible for the patients’ recruitment and data collection, namely, XG, XW, YTH, YYH, YY, and HuW. ZS, HaW, JL, and XG responsible for the patient’s treatment strategy. XG followed up the whole pregnant women and wrote the article and ZS guided and supervised the whole process. All authors contributed to the article and approved the submitted version.

## Funding

This study was funded by the National Natural Science Foundation of China (81970682, 81570709); the National Key Research and Development Program of China (2017YFC0907403); Health and Medicine Research Foundation in Shenyang City (17-230-9-02); The central government guided local special funds for scientific and technological development (2019416021).

## Conflict of Interest

The authors declare that the research was conducted in the absence of any commercial or financial relationships that could be construed as a potential conflict of interest.

## Publisher’s Note

All claims expressed in this article are solely those of the authors and do not necessarily represent those of their affiliated organizations, or those of the publisher, the editors and the reviewers. Any product that may be evaluated in this article, or claim that may be made by its manufacturer, is not guaranteed or endorsed by the publisher.
