# Supplementary material for: Postpartum Thyroid Dysfunction in Women With Known and Newly Diagnosed Hypothyroidism in Early Pregnancy
Source: Front Endocrinol (Lausanne). 2021 Nov 26;12:746329. doi: 10.3389/fendo.2021.746329 (PMC8662309; doi:10.3389/fendo.2021.746329)
Supplement: Supplementary file 1 [file Table_1.docx]

**Supplement Table 1.** summarizes the clinical characteristics and thyroid function tests of 49/50 (98.0%) women with ND-SCH by whether they remained euthyroid (n=18) or developed PPT (n=31) in the first year of postpartum

| **ND-SCH group** | **Euthyroidism** | **PPT** | **P** |
| --- | --- | --- | --- |
| **N (%) *** | 18/50 (36.0) | 31/50 (62.0) | 0.020* |
| **Maternal age (years)** | 31.33 ± 3.97 | 30.77 ± 4.14 | 0.646 |
| **Pre-pregnancy BMI (kg/m2)** | 21.56 ± 3.09 | 21.02 ± 3.08 | 0.559 |
| **GWG**≥**15 kg, n (%)** | 8 (44.4) | 18 (58.1) | 0.357 |
| **Family history of thyroid disease, n (%)** | 2 (11.1) | 8 (25.8) | 0.388 |
| **Delivery history, n (%)** | 1 (5.6) | 6 (19.4) | 0.364 |
| **Abortion history, n (%)** | 6 (33.3) | 9 (29.0) | 0.753 |
| **TSH level in BP (mU/L)** | 1.51 (0.73-3.05) | 1.68 (1.24-2.53) | 0.870 |
| **Diagnosis gestational age (weeks)*** | 12.0 (6.4-14.9) | 6.0 (5.0-9.0) | 0.001* |
| ≤8.0 weeks, n (%) * | 5 (27.8) | 23 (74.2) | 0.002* |
| **Initial TSH level in T1 (mU/L)** | 5.17 (4.34-6.33) | 5.66 (4.37-7.70) | 0.276 |
| **TSH level in T2 (mU/L)** | 2.33 (1.66-3.25) | 1.63 (1.15-2.93) | 0.054 |
| **TSH level in T3 (mU/L)** | 1.89 (1.25-2.78) | 1.48 (0.88-2.15) | 0.583 |
| <1 mU/L, n (%) * | 0 | 11 (35.5) | —— |
| 1-2.5 mU/L, n (%) * | 13 (72.2) | 13 (41.9) | 0.041* |
| ＞2.5 mU/L, n (%) | 5 (27.8) | 7 (22.6) | 0.708 |
| **TPOAb**≥**300μIU/mL, n(%)** | 3 (16.7) | 7 (22.6) | 0.899 |
| **TgAb**≥**300μIU/mL, n(%)** | 4 (22.2) | 9 (29.0) | 0.853 |
| **TPOAb/TgAb +, n (%)** | 5 (27.8) | 14 (45.2) | 0.229 |

*P<0.05, data were presented as the median (interquartile range: 25-75%), M±SD or n (%) as appropriate.

Abbreviation: PPT, postpartum thyroiditis; BMI, body mass index; GWG, gestational weight gain; TPOAb, thyroid peroxidase antibody; TgAb, thyroglobulin antibody; TSH, thyroid-stimulating hormone; BP: the period before pregnancy; T1, the first trimester of pregnancy; T2, the second trimester of pregnancy; T3, the third trimester of pregnancy.

**Supplement Table 2.** summarizes the clinical characteristics and thyroid function tests of 97/101 (96.0%) women with previously known AIT hypothyroidism (PK-SCH 51 & PK-OH 50) by whether they remained euthyroid (n=32) or developed PPT (n=65) in the first year of postpartum.

| **AIT group** | **Euthyroidism** | **PPT** | **P** |
| --- | --- | --- | --- |
| **N (%) *** | 32/101 (31.7) | 65/101 (64.4) | 0.000* |
| **Maternal age (years)** | 30.72 ± 3.30 | 30.25 ± 3.98 | 0.563 |
| **Pre-pregnancy BMI (kg/m2)** | 21.71 ± 2.98 | 22.44 ± 3.44 | 0.307 |
| **GWG**≥**15 kg, n (%)** | 20 (62.5) | 31 (47.7) | 0.170 |
| **Family history of thyroid disease, n (%)** | 2 (6.3) | 11 (16.9) | 0.257 |
| **Delivery history, n (%)** | 5 (15.6) | 9 (13.8) | 0.815 |
| **Abortion history, n (%)** | 12 (37.5) | 18 (27.7) | 0.326 |
| **TSH level in BP (mU/L)** | 2.59 (1.64-3.46) | 1.91 (0.91-3.04) | 0.155 |
| **TSH level in T1 (mU/L)** | 2.10 (1.74-3.12) | 3.20 (1.95-4.35) | 0.014* |
| >2.5 mU/L, n (%) * | 11 (34.4) | 43 (66.2) | 0.003* |
| **TSH level in T2 (mU/L)** | 1.66 (1.23-2.45) | 1.47 (0.83-2.33) | 0.535 |
| **TSH level in T3 (mU/L)** | 1.60 (0.92-2.51) | 1.59 (0.94-2.29) | 0.942 |
| <1 mU/L, n (%) | 10 (31.3) | 17 (26.2) | 0.599 |
| 1-2.5 mU/L, n (%) | 15 (41.9) | 37 (56.9) | 0.351 |
| ＞2.5 mU/L, n (%) | 7 (21.9) | 11 (16.9) | 0.555 |
| **TPOAb**≥**300μIU/mL, n(%) *** | 4 (12.5) | 32 (49.2) | 0.001* |
| **TgAb**≥**300μIU/mL, n(%)** | 7 (21.9) | 24 (36.9) | 0.853 |
| **TPOAb/TgAb +, n (%) *** | 9 (28.1) | 38 (58.5) | 0.005* |

*P<0.05, data were presented as the median (interquartile range: 25-75%), M±SD or n (%) as appropriate.

Abbreviation: PPT, postpartum thyroiditis; BMI, body mass index; GWG, gestational weight gain; TPOAb, thyroid peroxidase antibody; TgAb, thyroglobulin antibody; TSH, thyroid-stimulating hormone; BP: the period before pregnancy; T1, the first trimester of pregnancy; T2, the second trimester of pregnancy; T3, the third trimester of pregnancy.

**Supplement Table 3.** summarizes the clinical characteristics and thyroid function tests of 99/101 (98.0%) women with SCH (ND-SCH 50 & PK-SCH 51) by whether they restarted LT4 replacement (n=58) or not (n=41) in the first year of postpartum

| **SCH group** | **LT4 discontinuation** | **LT4 retreatment** | **P** |
| --- | --- | --- | --- |
| **N (%) *** | 41/101 (40.6) | 58/101 (57.4) | 0.016* |
| **Maternal age (years)** | 30.44±3.43 | 30.43±3.73 | 0.991 |
| **Pre-pregnancy BMI (kg/m2)** | 21.30±3.15 | 22.29±3.64 | 0.163 |
| **GWG>15 kg, n (%)** | 21 (51.2) | 29 (50.0) | 0.905 |
| **Family history of thyroid disease, n (%)** | 5 (12.2) | 10 (17.2) | 0.490 |
| **Delivery history, n (%)** | 2 (4.9) | 7/56 (12.1) | 0.384 |
| **Abortion history, n (%)** | 12 (29.3) | 19 (32.8) | 0.712 |
| **TSH level in BP (mU/L)** | 2.23 (1.43-3.30) | 1.73 (0.93-2.53) | 0.050 |
| **TSH level at <8 weeks in T1 (mU/L)** | 4.04 (2.37-5.37) | 4.35 (2.72-6.53) | 0.152 |
| >2.5 mU/L, n (%) * | 17 (41.5) | 37 (63.8) | 0.028* |
| **TSH level in T2 (mU/L)** | 1.96 (1.34-2.84) | 1.78 (1.20-2.93) | 0.529 |
| **TSH level in T3 (mU/L)** | 1.57 (1.17-2.52) | 1.59 (0.90-2.64) | 0.655 |
| <1 mU/L, n (%) * | 3 (7.3) | 19 (32.8) | 0.003* |
| 1-2.5 mU/L, n (%) * | 29 (70.7) | 24 (41.4) | 0.004* |
| ＞2.5 mU/L, n (%) | 9 (22.0) | 15 (25.9) | 0.655 |
| **TPOAb**≥**300μIU/mL, n(%) *** | 3 (7.3) | 20 (34.5) | 0.002* |
| **TgAb**≥**300μIU/mL, n(%)** | 10 (24.4) | 19 (32.8) | 0.367 |
| **TPOAb/TgAb +, n (%) *** | 11 (26.8) | 29 (50.0) | 0.021* |

*P<0.05, data were presented as the median (interquartile range: 25-75%), M±SD or n (%) as appropriate.

Abbreviation: BMI, body mass index; GWG, gestational weight gain; TPOAb, thyroid peroxidase antibody; TgAb, thyroglobulin antibody; TSH, thyroid-stimulating hormone; BP: the period before pregnancy; T1, the first trimester of pregnancy; T2, the second trimester of pregnancy; T3, the third trimester of pregnancy.
